# Supplementary material for: Molecular Mechanisms of Inhibitory Effects of Bovine Lactoferrin on Invasion of Oral Squamous Cell Carcinoma
Source: Pharmaceutics. 2023 Feb 7;15(2):562. doi: 10.3390/pharmaceutics15020562 (PMC9958951; doi:10.3390/pharmaceutics15020562)
Supplement: Supplementary file 1 [file pharmaceutics-15-00562-s001.zip › pharmaceutics-2133461-supplementary.pdf]

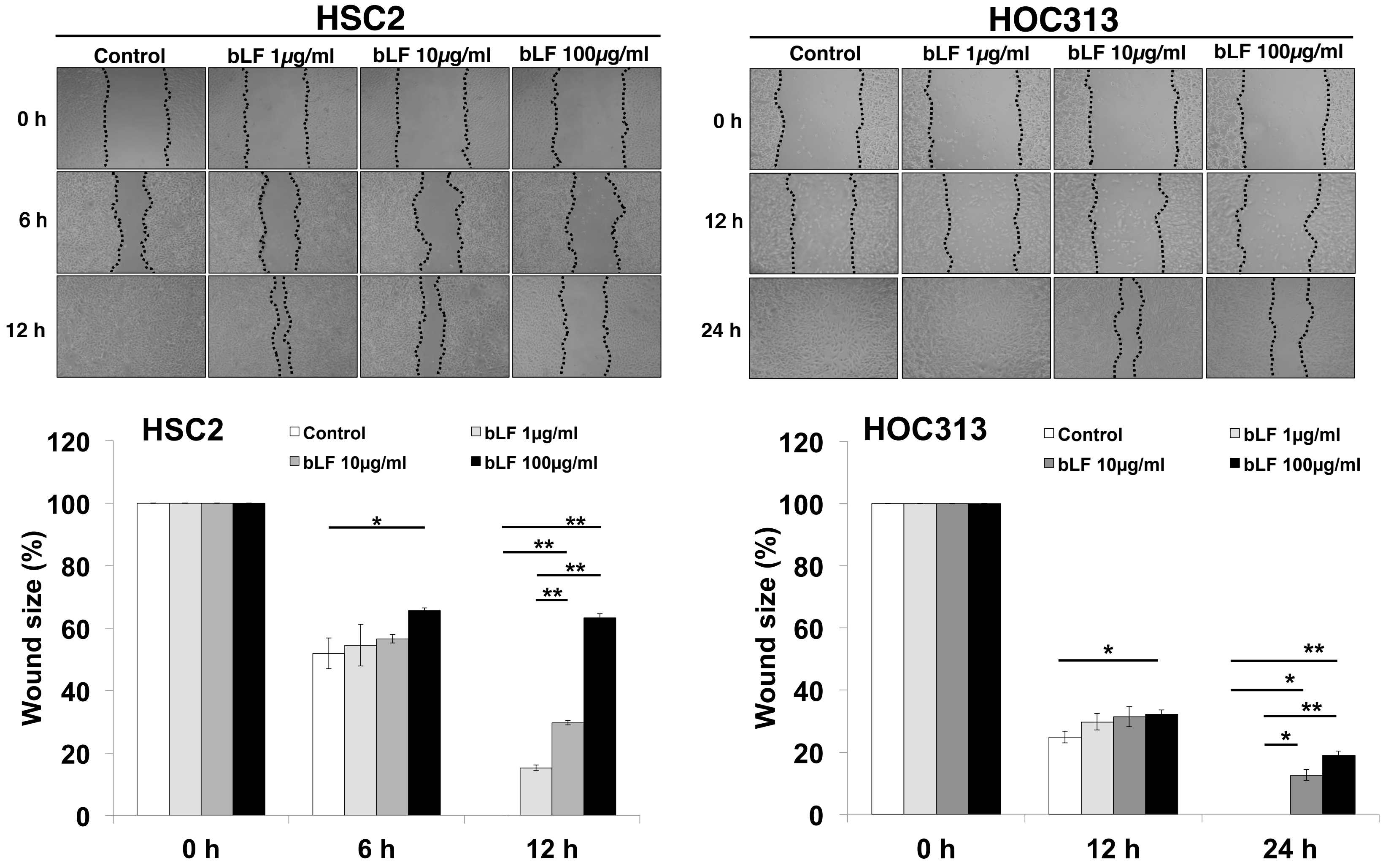

Supplementary Figure S1: Bovine lactoferrin inhibited migration of HSC2 and HOC313 cells.

Six hundred thousand HSC2 and HOC313 cells were cultured in 6 cm plates. The effects of bLF on migration of OSCC cells were investigated using a wound healing assay after pretreatment with 1, 10, or 100 μg/ml bLF. Photos were taken at 6 h and 12 h after scratching for HSC2 cells and 12 h and 24 h for HOC313 cells. Data (n = 3) represent the SD of triplicate experiments. * $p < 0.05$; ** $p < 0.01$.

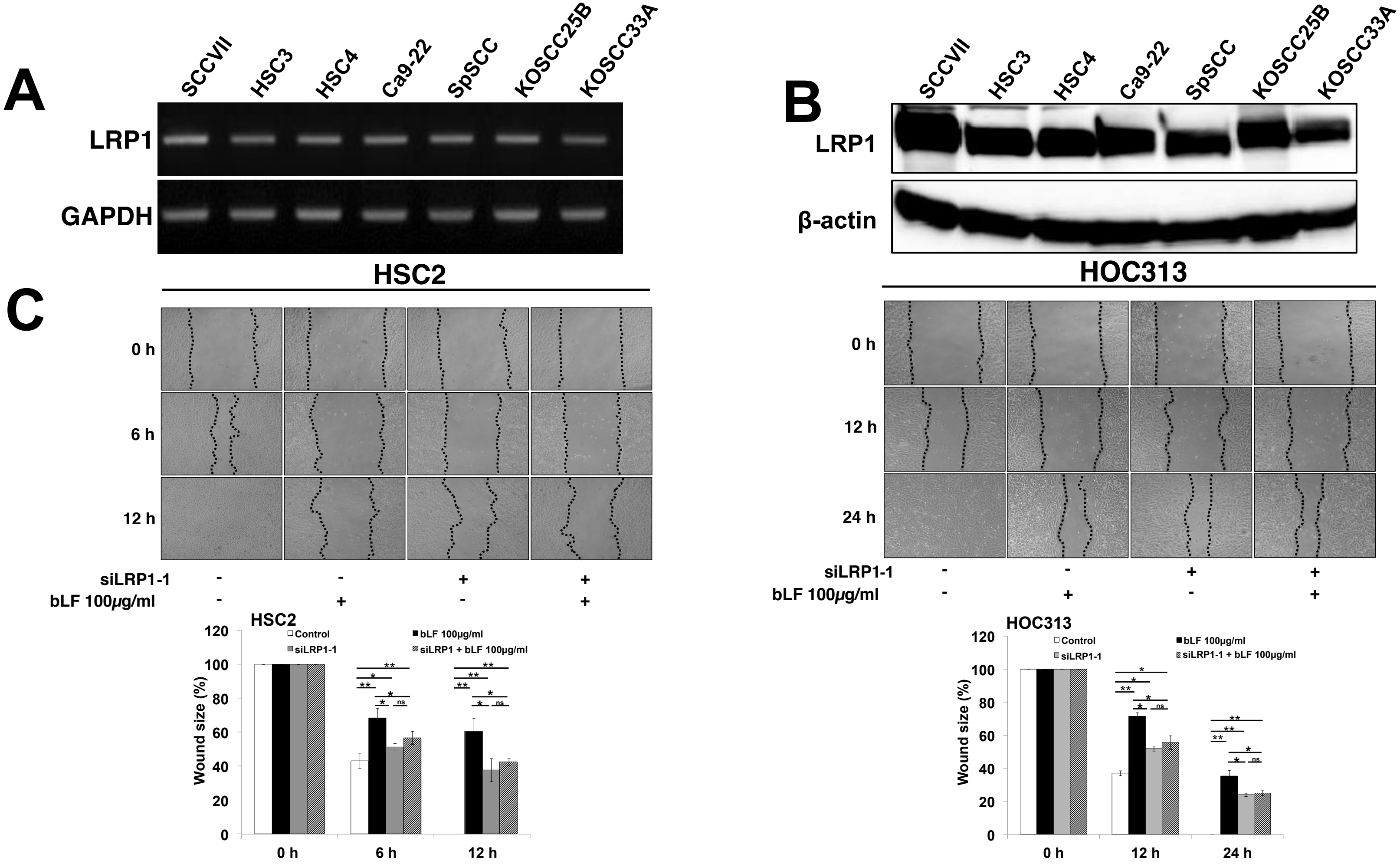

Supplementary Figure S2: Bovine lactoferrin suppressed migration of HSC2 and HOC313 cells through LRP1.

Seven OSCC cell lines were plated in 6 well-plates for mRNA analysis and 6 cm dishes for proteins. (A) and (B) All examined OSCC cell lines express LRP1. (C) The siLRP1-1 were transfected into HSC2 and HOC313 cells as described in the Materials and Methods. Cultured plates were scratched using 1 ml pipette tips treated with or without bLF (100 μg/ml). The gaps were observed and images were taken at 6 h and 12 h for HSC2 cells and 12 h and 24 h for HOC313 cells after bLF treatment. Typical data of 3 independent experiments (n = 3) are shown. * $p < 0.05$; ** $p < 0.01$. ns: not significant.

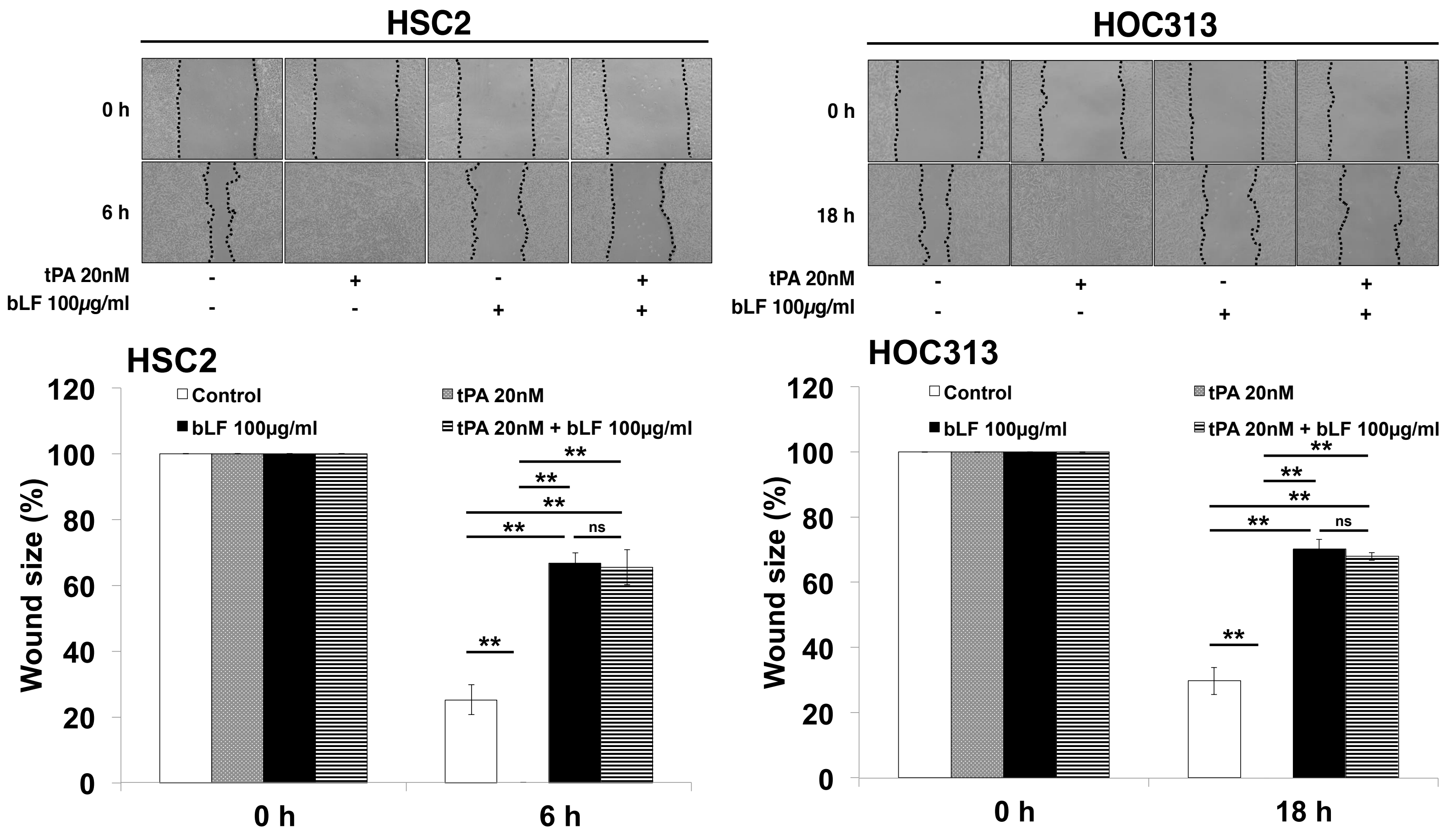

Supplementary Figure S3: Bovine lactoferrin attenuated tPA-induced HSC2 and HOC313 cell migration. tPA (20 nM) and bLF (100 µg/ml) were used as detailed in the Materials and Methods. Culture plates of HSC2 and HOC313 cells were scratched and stimulated in the presence of tPA (20 nM), bLF (100 µg/ml), or a mixture of tPA and bLF. Wound closure was measured at 6 h for HSC2 cells and 18 h for HOC313 cells after stimulation. The gaps were investigated at 18 h after treatments. n = 3 in triplicate. * $p < 0.05$; ** $p < 0.01$. ns: not significant.

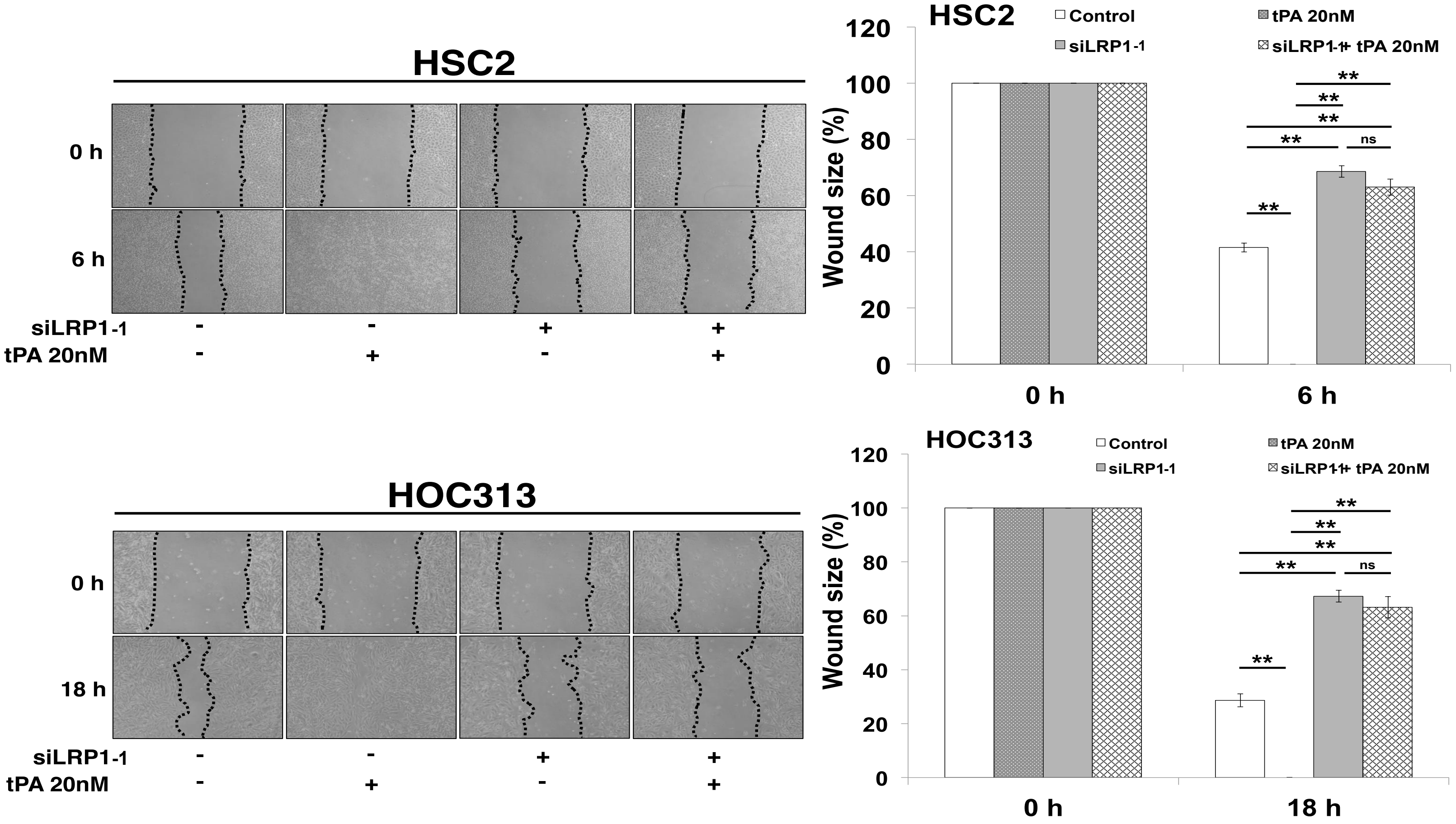

Supplementary Figure S4: Bovine lactoferrin decreased tPA-induced migration of OSCC cells through LRP1.

HSC2 and HOC313 cells were plated into 6-well-dishes. Cells were knocked down as described in the Materials and Methods. tPA was added after scratching, and wound healing was investigated at 6 h for HSC2 cells and 18 h for HOC313 cells. All experiments were performed three times (n = 3) and are presented as the mean ± S.D. Statistical analysis was performed using one-way ANOVA. $**p < 0.001$ compared with the control group. ns: not significant.

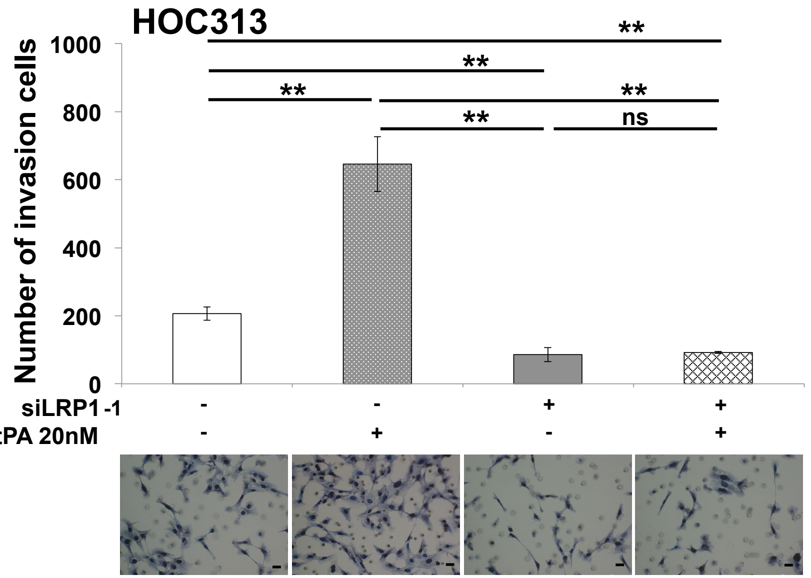

Supplementary Figure S5: Bovine lactoferrin repressed tPA-induced invasion of HOC313 cells through LRP1.

HOC313 cells were subjected to knockdown using siLRP1-1 and RNAiMAX as described in the Materials and Methods. Knockdown cells were then detached and seeded in chambers coated with Matrigel with and without tPA (20 nM) treatment. The invasive cells were finally fixed and stained. The number of invasive cells was analyzed and is presented as the mean ± S.D. Statistical analysis was performed using one-way ANOVA. $^{**}p < 0.001$ compared with the control group. ns: not significant. Scale bar 100 μm.
